# The Influence of the Microbiome on the Complications of Radiotherapy and Its Effectiveness in Patients with Laryngeal Cancer

**DOI:** 10.3390/cancers16213707

**Published:** 2024-11-01

**Authors:** Karolina Dorobisz, Tadeusz Dorobisz, Katarzyna Pazdro-Zastawny, Katarzyna Czyż, Marzena Janczak

**Affiliations:** 1 Department of Otolaryngology, Head and Neck Surgery, Wrocław Medical University, 50-367 Wroclaw, Poland; 2 Department of Vascular, General and Transplantation Surgery, Wroclaw Medical University, 50-367 Wroclaw, Poland; 3 Institute of Animal Breeding, Faculty of Biology and Animal Science, Wroclaw University of Environmental and Life Sciences, 50-375 Wroclaw, Poland

**Keywords:** head and neck cancer, microbiome, laryngeal cancer, radiotherapy, radiation-induced oral mucositis

## Abstract

Introduction: Radiotherapy is an effective method of treating cancer and affects 50% of patients. Intensity-modulated radiotherapy (IMRT) is a modernized method of classical radiation used in the treatment of laryngeal cancer. Treatment with intent to preserve the larynx is not always safe or complication-free. The microbiome may significantly influence the effectiveness of oncological treatment, especially radiotherapy, and may also be modified by the toxic response to radiation. Objective: The aim of the study was to prospectively assess the microbiome and its influence on radiotherapy toxicity in patients with laryngeal cancer. Results: Statistically significant risk factors for complications after radiotherapy were the percentage of Porphyromonas of at least 6.7%, the percentage of Fusobacterium of at least 2.6% and the percentage of Catonella of at least 2.6%. Conclusions: The importance of the microbiome in oncology has been confirmed in many studies. Effective radiotherapy treatment and the prevention of radiation-induced oral mucositis is a challenge in oncology. The microbiome may be an important part of personalized cancer treatment. The assessment of the microbiome of patients diagnosed with cancer may provide the opportunity to predict the response to treatment and its effectiveness. The influence of the microbiome may be important in predicting the risk group for radiotherapy treatment failure. The possibility of modifying the microbiome may become a goal to improve the prognosis of patients with laryngeal cancer. Fusobacterium, Porphyromonas and Catonella are important risk factors for radiation-induced oral mucositis in patients with laryngeal cancer.

## Simple Summary

The aim of the study was to prospectively assess the microbiome and its influence on radiotherapy toxicity in patients with laryngeal cancer. Statistically significant risk factors for complications after radiotherapy were the percentage of Porphyromonas of at least 6.7%, the percentage of Fusobacterium of at least 2.6% and the percentage of Catonella of at least 2.6%. The importance of the microbiome in oncology has been confirmed in many studies. Effective radiotherapy treatment and the prevention of radiation-induced oral mucositis is a challenge in oncology. The microbiome may be an important part of personalized cancer treatment. The assessment of the microbiome of patients diagnosed with cancer may provide the opportunity to predict the response to treatment and its effectiveness. The influence of the microbiome may be important in predicting the risk group for radiotherapy treatment failure. The possibility of modifying the microbiome may become a goal to improve the prognosis of patients with laryngeal cancer. Fusobacterium, Porphyromonas and Catonella are important risk factors for radiation-induced oral mucositis in patients with laryngeal cancer.

## 1. Introduction

Head and neck squamous cell carcinoma (HNSCC) ranks sixth among the world’s most common cancers, registering over half a million new cases worldwide each year, and the 5-year survival rate ranges from 25 to 60% [1,2]. In 2020, 184,615 new cases and 99,840 deaths due to laryngeal cancer were registered [3]. The risk factors for HNSCC are smoking, alcohol consumption, human papillomavirus (HPV) and male gender [4]. Data indicate that 15–20% of cancers are caused by infectious agents, 20–30% by smoking, and 30–35% by unhealthy lifestyle, diet, lack of physical activity and obesity [5]. Oncogenic viruses have been well known; their action is based on integration into the host genome and inactivation of tumor suppressor genes, such as p53 in the case of HPV infection [6,7]. In the treatment of HNSCC, combined therapies are used, consisting of surgery, radiotherapy, chemotherapy and immunotherapy, but the effects of this treatment are not satisfactory, and the survival rate is low.

Radiotherapy is an effective method of treating cancer; over 50% of oncological patients are treated with this method [8,9]. Intensity-modulated radiotherapy (IMRT) is a modernized method of classical radiation used in the treatment of laryngeal cancer. Adaptive radiotherapy (ART) provides the opportunity to optimize treatment, primarily by sparing critical organs and increasing the dose to the tumor area [10]. Treatment with the intent to preserve the larynx is not always safe or complication-free. Acute complications during radiotherapy affect the patient’s quality of life. Complications may cause discontinuation of therapy, which will result in a lack of treatment effectiveness and a worse prognosis for the patient. Toxic complications of radiotherapy include radiation-induced oral mucositis (RIOM), dermatitis, fibrosis and tissue necrosis [11]. The known risk factors include radiation dose, patient’s age, smoking and chronic diseases [12]. Biological factors have also been shown to be associated with the incidence of radiotherapy complications, causing radiotherapy resistance, hypoxia, inflammation and immune system dysfunction [13].

T1, T2 and T3 laryngeal cancers in the early stages can be treated with radiotherapy or surgery. For tumors requiring total laryngectomy (T3), an organ preservation strategy of neoadjuvant chemotherapy followed by radiotherapy or chemoradiotherapy is recommended. For T4 tumors, total laryngectomy followed by radiotherapy with chemotherapy is recommended, if possible.

Next-generation sequencing (NGS) has allowed us to change our view of the bacterial world. The microbiome consists of the genes and genome of the microbiota, as well as products of the host’s microflora, such as plasmid DNA, viruses, fungi and archaea [14]. The human microbiome is individually variable, and its composition may be primarily influenced by environmental factors and the host [15,16]. Many studies are currently analyzing the functions of the microbiome in the pathogenesis of many diseases, and its relationship with inflammatory diseases, obesity, allergy, autism, depression and cancer has been proven [17,18,19]. Dysbiosis is a microbiome imbalance; it promotes oncogenesis by intensifying inflammatory processes and affecting the host’s metabolism. The microbiome plays a very important role in the functioning of the immune system [20,21].

The microbiome may significantly influence the effectiveness of oncological treatment, especially radiotherapy, and may also be modified by the toxic response to radiation.

Profiling the microbiome in various types of cancer is analyzed in many studies. However, there is still little information on the correlation of the microbiome with HNSCC and its impact on treatment effects. New treatment options that may be provided by expanding knowledge on this topic are very important for improving the treatment outcomes of patients with laryngeal cancer and creating personalized medicine.

The aim of this study was to prospectively assess the microbiome and its influence on radiotherapy toxicity in patients with laryngeal cancer.

## 2. Material and Method

### 2.1. Study Group

The study included a group of 40 patients treated for squamous cell carcinoma of the larynx who received radiotherapy (7 women and 33 men), aged from 42 to 80 years (mean M = 63.9, SD = 9.1). The study was conducted in the Department of Otolaryngology of the University Clinical Hospital in Wrocław, Poland. Patients were enrolled in the study consecutively. The study was conducted in 2022–2023. Inclusion criteria for the study included patients with squamous cell carcinoma of the larynx treated with radiotherapy as a single or combined method. The exclusion criteria for the study included patients with chronic inflammation of the upper and lower respiratory tract, treatment with antibiotics in the last 6 months, a history of other cancers, and acute infections. The patients were divided into two groups—patients who did not present any significant complications during radiotherapy and within 6 months after treatment, and a group of patients who developed RIOM during radiotherapy treatment that resulted in a delay or inability to complete the treatment course.

Each patient underwent diagnostic imaging, a tumor biopsy was taken, and then the disease was classified according to TNM staging, on the basis of which a decision on the patient’s treatment method was made. The general condition of the patients was assessed according to the ECOG scale. Laboratory tests were analyzed in each patient, including blood count and nutritional parameters, such as total protein, total cholesterol, HDL, LDL, iron level, TSH and CRP protein. The nutritional status of patients was assessed, qualifying patients as satisfactorily nourished, at risk of malnutrition or with malnutrition. Then, a swab was taken from each patient for microbiological culture and a swab to assess the microbiome. Microbiome assessment was performed using 16S rRNA sequencing.

### 2.2. Microbiome Profiling

1. DNA isolation from cotton swab samples using a commercial kit following the manufacturer’s protocol (GeneMATRIX Swab-Extract DNA Purification Kit, Eurx, Gdańsk, Poland).
2. Quality control of isolated DNA—concentration and purity evaluation (Qubit 4 Fluorometer, Invitrogen, St. Bend, OR, USA and DeNovix DS-11 spectrophotometer, West Haven, Connecticut, USA); DNA integrity check by electrophoresis on 1.5% agarose gel.
3. Amplifier library construction after rounds of PCR amplification.
  - Amplification of specific target DNA region of bacterial 16S ribosomal RNA (V3–V4) using universal primers connected with Illumina sequencing adapters; PCR Clean-Up using AMPure XP beads, Beckman Coulter, Inc., Indianapolis, IN, USA.
  - Index PCR attaching dual indices and Illumina sequencing adapters using the Nextera XT Index Kit; PCR Clean-Up using AMPure XP beads, Indianapolis, IN, USA.
4. Library QC, quantification, normalization and pooling.
5. Sequencing on MiSeq–Using paired 300 bp reads.

### 2.3. Statistical Analysis

The analysis was done using the Statistica v. 13.3 package (TIBCO Software Inc., Palo Alto, CA, USA). A significance level of alpha smaller than or equal to 0.05 was used. Continuous variables were reported as mean values and standard deviation or, if not normally distributed, as median and interquartile range, while categorical variables were reported as percentages. Continuous variables were compared by *t*-test or Mann–Whitney U-test (for non-parametric variables), while categorical variables were compared by Fisher’s exact test. Multiple logistic regression analyses were performed to identify independent predictors of complications following radiotherapy. The area under the ROC curve was used to evaluate the predictive models.

## 3. Results

Patients in the compared groups did not differ significantly in terms of all analyzed sociodemographic and somatic characteristics (*p* < 0.05). The majority of the study group was male, most of them had primary or secondary education and 37.5% of patients were single. Basic statistics characterizing patients with different complications after radiotherapy are presented in Table 1.

There were no differences in the general condition of patients between the groups; however, the condition of the entire study group was classified as ECOG 1 in 70% and ECOG 2 in 5%. Patients without complications were more likely to report swallowing disorders before treatment. For this reason, they were often fed through a gastrostomy, which may have prevented complications. However, patients who experienced RIOM were more likely to suffer from chronic diseases (62.5% vs. 40.6%). Tobacco smoking and alcohol consumption were comparable in both groups, and it concerned the entire group of patients—97.5% of patients reported smoking tobacco and 57.5% alcohol consumption. Periodontal diseases were more common in patients from the group with RIOM (87.5% vs. 65.6%), but the differences were not statistically significant. Nutritional status also did not differ significantly between groups. The results of the medical interview and survey in the group with and without RIOM are presented in Table 2.

RIOM was not statistically significantly related to the location of the tumor; it occurred equally often in patients with glottic and subglottic tumors. The stage of the cancer and the type of treatment—stand-alone or adjuvant radiotherapy—did not differ statistically significantly between the groups. These data are presented in Table 3.

The tests performed on swabs and classic cultures showed no statistically significant differences. However, in both groups, pathogenic bacteria not belonging to the physiological flora were detected, such as *Pseudomonas*, *Klebsiella*, *Enterobacter*, *Serratia*, *Escherichia* or fungi of the *Candida* genus. Bacteria detected in cultures are presented in Table 4.

The tests performed to assess the microbiome of the mucosa around the tumor revealed significant statistical differences regarding *Porphyromonas*, *Fusobacteroium*, *Gemella* and *Catonella*. These bacteria were statistically more common in patients with RIOM compared to the group of patients without complications. The data are shown in Table 5.

**Figure 1:**
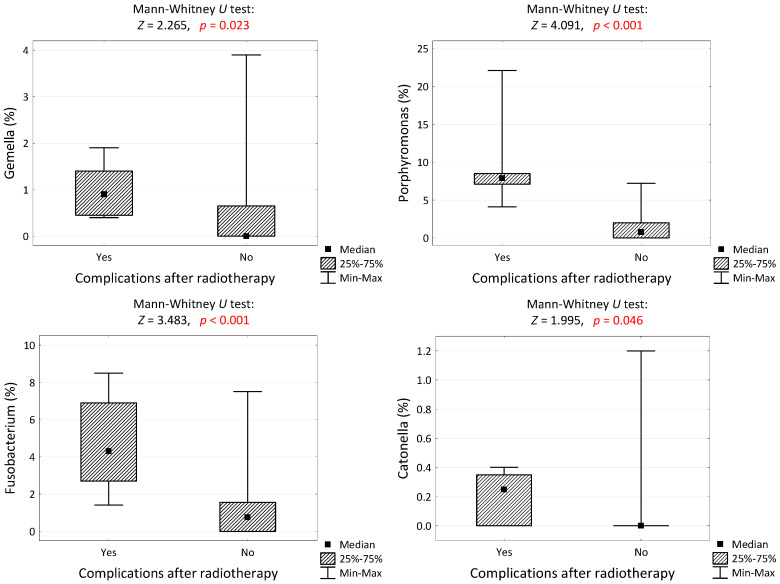
The percentage of bacteria of the genus *Gemella*, *Porphyromonas*, *Fusobacterium* and *Catonella* in the group of patients with and without complications after radiotherapy and the results of the significance test. Red color means that the difference is statistically significant.

The occurrence of complications after radiotherapy correlates with the percentage of isolated bacteria of the genera *Porphyromonas*, *Fusobacterium*, *Gemella* and *Catonella* (Figure 1).

ROC curves were used to establish thresholds for distinguishing patients with complications after radiotherapy. Cut-off values, test sensitivity and specificity, and the area under the curve were estimated for four bacteria (Table 6).

Statistically significant risk factors for complications after radiotherapy were the percentage of *Porphyromonas* bacteria of at least 6.7%, the percentage of *Fusobacterium* bacteria of at least 2.6% and the percentage of *Catonella* bacteria of at least 2.6%.

Logistic regression analysis was used to determine independent predictors of complications, taking into account all four risk factors. *Porphyromonas* and *Fusobacterium* bacteria turned out to be independent predictors of complications after radiotherapy (Table 7).

The model for estimating the probability of complications takes the logit form

logit Pr {Complications = 1|X} = −7.17 + 0.860 × *Porphyromonas* + 0.521 × *Fusobacterium*

The risk of complications in patients with at least 6.7% *Porphyromonas* was twenty-eight times higher compared to patients with a lower percentage of these bacteria (RR = 28.0). The risk of complications in patients with 2.6% or more *Fusobacterium* isolated is twenty-one times higher compared to patients with a lower percentage of these bacteria (RR = 21.0). The risk of complications in patients in whom at least 0.2% *Catonella* was isolated is five times higher compared to patients with a lower percentage of these bacteria (RR = 5.0). The data are presented in Table 8.

## 4. Discussion

The microbiome plays an important role in oncogenesis, the course of the disease and the effectiveness of cancer treatment [22,23]. The impact of the microbiome on the course and effects of radiotherapy may be crucial for improving the effectiveness of this treatment, but there are few publications discussing this topic [24].

The relationship between cancer treatment and the microbiome may result from a primary disturbance in the microbiome of the cancerous tissue or from the impact of cancer treatment on the microbiome. Most studies on the microbiome use 16S rRNA sequencing with analysis of taxonomic distribution and assessment of microbiome diversity, and this method was also used in the discussed study. The diversity of the microbiome of cancer tissues promotes a good prognosis and a better survival rate [25]. In the gastrointestinal tract, radiotherapy causes changes in the diversity of the microbiome [15,26,27,28]. It has been proven that the intestinal microbiome influences the effectiveness of surgery, chemotherapy, radiotherapy and immunotherapy [18,20,29].

Cancer treatment is a challenge of 21st-century medicine. Inflammation is very important in the process of cancer development and is responsible for angiogenesis and metastasis. Dysbiosis affects the local and systemic immune response. The microbiome is modified by external factors such as diet, drug use, smoking and alcohol consumption; its composition also depends on the immune system and the genetic susceptibility of the host [30]. The gastrointestinal microbiome and its impact on many diseases have been understood, including the course and response to cancer treatment. Touchefeu et al. presented the results of stool analysis of 45 patients; they proved that before the initiation of treatment, the response to chemoradiotherapy in the intestinal microbiome of patients with an increased amount of *Bacteroides* was worse than in other patients [31]. Yu et al. proved the important function of *Fusobacterium* in the occurrence of chemoresistance in colorectal cancer by activating autophagy [32]. *Fusobacterium* is an anaerobic bacterium associated with periodontal diseases; it provokes inflammation and may be responsible for oncogenesis, and the progression of colorectal cancer in the presence of this bacterium has been confirmed [33,34]. The occurrence of an increased amount of *Fusobacterium* has also been associated with cancer recurrences [35]. It has also been confirmed that changes in the microbiome are a factor causing radiation enteropathy [36].

Radiotherapy and radiochemotherapy, whether or not they are combined with surgery, are the mainstay of treatment for HNSCC. Radiation damages tissues, which increases inflammation, allowing microorganisms to penetrate inside the mucous membrane. RIOM is the most common complication of radiotherapy for HNSCC. It leads to a deterioration in quality of life and may cause a delay in treatment, discontinuation of treatment or a need to reduce the radiation dose, which worsens the prognosis [37,38]. RIOM is a significant problem in oncological treatment; it causes pain, requiring the use of opioids in over 50% of cases, as well as malnutrition, increasing the risk of infection and the need for hospitalization [39,40]. RIOM is a common effect when radiotherapy is combined with surgery, especially influencing reconstructive outcomes [41]. It is often associated with depression and anxiety [42].

Studies on RIOM have not established treatment recommendations that significantly influence the prevention of its occurrence and modify its course [43]. Tao et al. present that the incidence of complications after radiotherapy depends on the stage of the cancer and the location and type of treatment, but according to this analysis, in laryngeal cancer, the location and stage of advancement were not significant [44]. According to another study, the incidence was associated with local metastases to the lymph nodes [45]. Smoking is associated with a more frequent occurrence and worse course of RIOM, but it is difficult to confirm it because most patients with HNSCC are smokers [46,47]. Other factors associated with a higher risk of complications of radiotherapy include dental condition, malnutrition, diabetes, kidney disease and older age [48,49,50]. The oral microbiome depends on pH, diet and tissue oxygenation [51,52,53]. Dysbiosis causes disturbances in the composition of saliva and affects periodontal diseases [54,55]. The microbiome influences the course of RIOM through inflammatory cytokines, which exacerbate inflammation, resulting in increased pain. The microbiome described during RIOM includes *Actinobacillus*, *Prevotella*, *Fusobacterium*, *Treponema*, *Porphyromonas*, *Capnocytophyga*, *Neisseria*, *Parviromonas*, *Olsenella* and *Candida* [56]. Zhu et al. [57] found an increase in Gram-negative bacteria after radiotherapy, while *Actinobaccilus* predominated in patients with complications. Vesty et al. [58] found that RIOM is associated with the presence of *Capnocytophyga*, *Olsenella* and *Parviomonas* before treatment. In our analysis, *Fusobacterium* and *Porphyromonas*, as well as *Catonella* and *Gemella,* predominated in patients with severe RIOM. The role of *Fusobacterium* is important, as its influence on the course of colorectal cancer and the effects of treatment has already been widely discussed in the literature. Despite the lack of complete evidence, publications and studies indicate that the composition of the microbiome influences the complications of radiotherapy [56,57,59,60].

Another study confirming this thesis showed in a mouse model that radiotherapy of pathogen-free mucous membranes does not cause complications in the form of RIOM [61]. Although IMRT significantly improved the treatment results and reduced side effects after radiotherapy, according to Shuurhuis et al., the microbiome does not differ when using the classic method or IMRT; therefore, IMRT did not affect the frequency of RIOM [62]. Dong et al. found that the administration of *Fusobacterium* resulted in a poorer response of the anticancer effect of radiotherapy [63]. Most likely, *Fusobacterium* promotes the levels of Ki-67, VEGF and CXCL1 proteins in cancer tissues. It has been shown that the accumulation of *Fusobacterium* in the oral cavity deteriorates the integrity of the intestinal epithelium and increases the risk of radiotherapy for colorectal cancer. In this study, mice were administered metronidazole, which improved the course of intestinal inflammation and also reduced tumor size and the levels of Ki-67, VEGF and CXCL1 proteins [63]. The microbiome is interconnected; changes in the oral cavity cause changes in the lower gastrointestinal tract [63]. However, oral dysbiosis worsens the effect of radiotherapy in the treatment of colorectal cancer [63].

The treatment of RIOM is primarily prevention, followed by opioid analgesics—morphine and fentanyl [64,65]. Prevention involves proper hygiene and the prevention of periodontal disease. In the course of RIOM, it is recommended to use steroids topically or generally [64]. Sayed et al. showed a positive effect of pentoxifylline and vitamin E on the reduced incidence of severe RIOM [66]. Malnutrition has negative effects related to healing and inflammation and weakens the immune system [49]. Many studies have shown that early nutritional interventions improve the prognosis of HNSCC patients treated with radiotherapy [67,68]. However, in a review paper assessing the literature on the impact of early nutritional intervention on the occurrence of RIOM, it was concluded that more detailed research is still necessary, especially regarding patients treated with radiotherapy [69]. In our analysis, patients who did not suffer from RIOM used gastrostomy more often. The modification of the microbiome with antibiotics showed that vancomycin influences a better immune response by reducing the number of bacteria sensitive to vancomycin; the effect of radiotherapy treatment was better after the use of antibiotics [70]. Cui et al. [71] confirmed a better response to radiotherapy in a group of mice treated with antibiotics that selectively eliminated specific groups of bacteria. Bullman et al. [72] also showed that treatment with metronidazole reduces the number of *Fusobacterium*, as well as the rate of tumor progression. Antibiotics modify the microbiome; their action and how they promote certain types of bacteria and inhibit the development of others is not fully clear in the pathogenesis of cancer, and they may be key to regulating dysbiosis, but they can also intensify it.

Therapy with probiotics—live microorganisms—has anti-inflammatory effects and, according to the authors, also anticancer effects [71]. The most frequently mentioned probiotics include *Lactobacillus*, *Bifidobacteria*, *Saccharomyces loulardii* and *Bacillus coagulans* [73,74]. Ho et al. showed that tumor regression was achieved in mice receiving probiotics and a proper diet [75]. Probiotics such as *Bifidobacterium*, *Lactobacillus* and *Streptococcus* promote microbiome diversity [51]. It has been shown that probiotics have a positive effect on the occurrence and severity of RIOM [76]. However, authors’ opinions on the use of probiotics differ; according to some, they do not reduce the complications of the surgical treatment of colorectal cancer and inflammation of the mucous membrane [77,78]. In the treatment of post-radiation enteritis, the modification of microflora using the FMT method is used, transferring microflora from healthy donors to the gastrointestinal tract of patients. Studies have shown that the microbiome of colorectal cancer patients promotes tumor formation in a mouse model, while the gastrointestinal microbiome of healthy donors inhibits tumor formation [79,80]. It has been confirmed that FMT reduced the symptoms of enteropathy and the toxicity of anticancer treatment.

There are also proposals for bioengineering of the microbiome—the use of genetically modified probiotics or bacteriophages [81,82,83]. The modification of the microbiome and regression of dysbiosis opens new possibilities in the treatment and prevention of head and neck cancer.

Improving the prognosis and effectiveness of radiotherapy treatment depends on the reason for differences in response to treatment in different patients. It is known that diet and the presence of chronic diseases influence complications after radiotherapy, but this does not give us a sufficient answer to many questions. The concept of the microbiome influencing the effects of radiotherapy is very interesting, but the mechanisms of its action are still unclear, and the number of original publications is insufficient. Radiotherapy is used as a stand-alone treatment method or in combination with chemotherapy or immunotherapy, but the synergism of the effect of these combinations, or the lack thereof, is also not fully explained.

### Limitations of the Study and Suggestions for Future Research

This work is based on a small number of patients. It is worth conducting a large clinical study assessing the impact of the microbiome on the effectiveness of oncological treatment in patients with HNSCC, taking into account the impact of other risk factors and modifications of the microbiome with various groups of antibiotics. It is possible that radiotherapy affects the composition of the microbiome during treatment. It would be necessary to collect samples to assess the microbiome after treatment. The current study aimed to assess the influence of the composition of the patient’s microbiome before radiotherapy on the risk of developing RIOM.

## 5. Conclusions

The importance of the microbiome in oncology has been confirmed in many studies. Effective radiotherapy treatment and the prevention of RIOM is a challenge in oncology. The microbiome may be an important part of personalized cancer treatment. Assessment of the microbiome of patients diagnosed with cancer may provide the opportunity to predict the response to treatment and its effectiveness. The influence of the microbiome may be important in predicting the risk group for radiotherapy treatment failure. The possibility of modifying the microbiome may become a goal to improve the prognosis of patients with HNSCC. *Fusobacterium*, *Porphyromonas* and *Catonella* are important risk factors for RIOM in patients with laryngeal cancer treated with radiotherapy.

## Figures and Tables

**Table 1:** General characteristics of patients in the group with and without complications after radiotherapy—RIOM and the results of significance and independence tests.

Variable	RIOM		*p*-Value
	Yes *n* = 8	No *n* = 32	
Gender:			0.309 ^a^
Male, *n* (%)	8 (100.0)	25 (78.1)	
Female, *n* (%)	0 (0.0)	7 (21.9)	
Age (years), M ± SD	62.8 ± 8.8	64.2 ± 9.3	0.696 ^b^
Education:			0.163 ^a^
Primary, *n* (%)	3 (37.5)	10 (31.3)	
Secondary, *n* (%)	4 (50.0)	18 (56.2)	
Incomplete higher, *n* (%)	1 (12.5)	0 (0.0)	
Higher, *n* (%)	0 (0.0)	4 (12.5)	
Place of residence:			0.727 ^a^
Village, *n* (%)	1 (12.5)	8 (25.0)	
Town up to 20,000, *n* (%)	2 (25.0)	4 (12.5)	
21–50,000 inhabitants, *n* (%)	1 (12.5)	6 (18.8)	
Over 50,000, *n* (%)	4 (50.0)	14 (43.7)	
Economic zone/urban area:			1.000 ^a^
Yes, *n* (%)	5 (62.5)	19 (59.4)	
No, *n* (%)	3 (37.5)	13 (40.6)	
Marital status:			0.230 ^a^
Singles, *n* (%)	3 (37.5)	12 (37.5)	
Partner/married relationship, *n* (%)	5 (62.5)	12 (37.5)	
With family support, *n* (%)	0 (0.0)	8 (25.0)	
BMI (kg/m^2^), Me [Q1–Q3]	23.3 [22.7–24.6]	22.3 [21.3–24.2]	0.161 ^c^

*n*—number, (%)—percentile, M—mean, SD—standard deviation, Me—median, Q1–Q 3—lower and upper quartile, *p*—test significance level, ^a^—Fisher’s exact test, ^b^—Student’s *t*-test, ^c^—Mann–Whitney U-test.

**Table 2:** Number (percentage) of people differing in the presence of complications—RIOM and analyzed clinical features, as well as the results of independence and significance tests.

Variable	RIOM-Yes *n* = 8	RIOM-No *n* = 32	*p*-Value
ECOG scale (score):			0.547
0—asymptomatic, n (%)	3 (37.5)	7 (21.9)	
1—symptomatic but completely ambulatory, *n* (%)	5 (62.5)	23 (71.9)	
2—Symptomatic, <50% in bed during the day, *n* (%)	0 (0.0)	2 (6.2)	
Swallowing disorders (yes)	2 (25.0)	13 (40.6)	0.686
Percutaneous endoscopic gastrostomy (yes)	1 (12.5)	9 (28.1)	0.653
Chronic diseases (yes)	5 (62.5)	13 (40.6)	0.430
Tuxedo (yes)	8 (100.0)	31 (96.9)	1.000
Drinking alcohol regularly (yes)	4 (50.0)	19 (55.4)	0.702
Dental conditions:			0.335
1—Normal, *n* (%)	0 (0.0)	7 (21.9)	
2—Cavities, caries, periodontal diseases, *n* (%)	7 (87.5)	21 (65.6)	
3—Edentulism, *n* (%)	1 (12.5)	4 (12.5)	
Nutritional status :			0.440
1—Satisfactory, *n* (%)	2 (25.0)	5 (15.6)	
2—Risk of malnutrition, *n* (%)	3 (37.5)	7 (21.9)	
3—Malnutrition, *n* (%)	3 (37.5)	20 (62.5)	

**Table 3:** Clinical characteristics of head and neck cancer patients treated with radiotherapy.

Clinical Parameters	RIOM		*p*-Value
	Yes *n* = 8	No *n* = 32	
Tumor location:			1.000
Glottis, *n* (%)	6 (75.0)	23 (71.9)	
Epiglottis, *n* (%)	2 (25.0)	9 (28.1)	
Tumor:			0.624
Tx, *n* (%)	1 (12.5)	4 (12.5)	
T1a, *n* (%)	0 (0.0)	5 (15.6)	
T1b, *n* (%)	4 (50.0)	12 (37.5)	
T2, *n* (%)	3 (37.5)	8 (25.0)	
T3, *n* (%)	0 (0.0)	3 (9.4)	
Node:			0.975
N0, *n* (%)	5 (62.5)	16 (50.0)	
N1, *n* (%)	1 (12.5)	4 (12.5)	
N2a, *n* (%)	0 (0.00)	1 (3.1)	
N2b, *n* (%)	1 (12.5)	6 (18.8)	
N2c, *n* (%)	1 (12.5)	4 (12.5)	
N3a, *n* (%)	0 (0.00)	1 (3.1)	
Stage:			0.605
I, *n* (%)	1 (12.5)	9 (28.1)	
II, *n* (%)	2 (25.0)	6 (18.8)	
III, *n* (%)	3 (37.5)	5 (15.6)	
IVa, *n* (%)	1 (12.5)	4 (12.5)	
IVb, *n* (%)	1 (12.5)	8 (25.0)	
Cervical lymph node groups *:			
I, *n* (%)	0 (0.0)	1 (3.1)	1.000
II, *n* (%)	3 (37.5)	16 (50.0)	0.698
III, *n* (%)	1 (12.5)	13 (40.6)	0.222
IV, *n* (%)	0 (0.0)	3 (9.4)	1.000
Not applicable, *n* (%)	5 (62.5)	16 (50.0)	0.698
Treatment:			0.333
Radiotherapy, *n* (%)	3 (37.5)	16 (50.0)	
Surgery + radiotherapy, *n* (%)	2 (25.0)	2 (6.2)	
Chemotherapy + radiotherapy, *n* (%)	2 (25.0)	5 (15.6)	
Surgery + chemotherapy + radiotherapy, *n* (%)	1 (12.5)	9 (28.2)	

* Multiple choice question; percentages do not add up to 100.

**Table 4:** Number (percentage) of patients differing in complications after radiotherapy—RIOM and isolated types of bacteria and fungi, as well as results of tests of independence (Fisher’s exact test).

Culture Result—Genus (Positive)	RIOM		*p*-Value
	Yes *n* = 8	No *n* = 32	
*Streptococcus oralis*, *n* (%)	3 (37.5)	12 (37.5)	1.000
*Staphylococcus aureus*, *n* (%)	0 (0.0)	3 (9.4)	1.000
*Candida albicans*, *n (%)*	2 (25.0)	15 (46.9)	0.428
*Neisseria*, *n* (%)	0 (0.0)	4 (12.5)	0.566
*Pseudomonas*, *n* (%)	0 (0.0)	5 (15.6)	0.563
*Serratia mercescens*, *n* (%)	0 (0.0)	3 (9.4)	1.000
*Bifidobacterium longum*, *n* (%)	1 (12.5)	1 (3.1)	0.364
*Corynebacterium*, *n* (%)	0 (0.0)	1 (3.1)	1.000
*Enterococcus faecalis*, *n* (%)	0 (0.0)	1 (3.1)	1.000
*Klebsiella*, *Enterobacter and Serratia*, *n* (%)	1 (12.5)	2 (6.2)	0.498
*Citrobacter freundii*, *n* (%)	0 (0.0)	1 (3.1)	1.000
*Lacticaseibicillus paracasei*, *n* (%)	0 (0.0)	2 (6.2)	1.000
*Morganella morganii*, *n* (%)	1 (12.5)	1 (3.1)	0.364
*Streptococcus dysgalactiae*, *n* (%)	0 (0.0)	1 (3.1)	1.000
*Veillonella parvula*, *n* (%)	1 (12.5)	0 (0.0)	0.200
*Escherichia coli*, *n* (%)	0 (0.0)	1 (3.1)	1.000
Absent, *n* (%)	4 (50.0)	5 (15.6)	0.059

**Table 5:** Descriptive statistics (median and interquartile range) of the percentage of isolated bacteria and fungi in groups of patients differing in the occurrence of complications after radiotherapy—RIOM and the results of significance tests (Mann–Whitney U-test).

Culture Result—Genus (%)	RIOM		*p*-Value
	Yes *n* = 8	No *n* = 32	
*Streptococcus*	5.1 [4.8–8.1]	7.6 [4.1–9.8]	0.636
*Prevotella melaninogenica*	16.2 [11.2–19.0]	11.3 [2.8–18.7]	0.287
*Prevotella*	18.6 [12.7–23.2]	14.3 [6.8–25.7]	0.748
*Rothia micilaginosa*	4.4 [2.1–6.5]	3.2 [1.4–8.3]	0.697
*Aggregatibacter*	0.0 [0.0–0.6]	0.0 [0.0–0.2]	0.839
*Gemella*	0.9 [0.5–1.4]	0.0 [0.0–0.6]	0.023
*Porphyromonas*	7.9 [7.1–8.5]	0.8 [0.0–2.0]	<0.001
*Fusobacterium*	4.3 [2.7–6.9]	0.8 [0.0–1.6]	<0.001
*Firmicutes*	0.2 [0.0–0.3]	0.0 [0.0–0.2]	0.319
*Corynebacterium matruchotii*	0.0 [0.0–1.2]	0.0 [0.0–0.4]	0.800
*Neiseria*	6.8 [0.4–9.9]	1.4 [0.1–6.0]	0.352
*Lacto bacillales*	2.4 [1.9–2.9]	3.9 [1.5–6.3]	0.176
*Actinobacteria*	1.9 [1.1–2.8]	2.1 [0.0–5.5]	0.852
*Actinomyces*	2.4 [1.5–4.6]	3.8 [1.0–6.6]	0.735
*Haemophilus*	5.8 [3.8–10.9]	2.7 [0.0–11.1]	0.335
*Capnocytophaga granulosa/gingivalis*	0.8 [0.3–2.2]	0.0 [0.0–1.0]	0.124
*Clostridiales*	0.7 [0.5–0.8]	0.3 [0.0–0.8]	0.193
*Veilonella*	0.0 [0.0–0.1]	0.0 [0.0–0.2]	0.946
*Campylobacter*	1.9 [1.3–2.7]	1.8 [0.0–3.8]	0.723
*Granulicatella*	0.0 [0.0–0.6]	0.0 [0.0–0.0]	0.302
*Lautropia*	0.0 [0.0–2.5]	0.0 [0.0–1.2]	0.852
*Shaalia odontolytica*	1.2 [0.9–2.4]	0.4 [0.0–3.0]	0.565
*Leptotricha*	0.9 [0.6–2.0]	0.5 [0.0–1.8]	0.187
*Stomatobaculum longum*	0.3 [0.1–0.4]	0.2 [0.0–0.5]	0.774
*Tannerell*	0.0 [0.0–0.2]	0.0 [0.0–0.3]	0.826
*Pasteurellaceae*	0.4 [0.2–0.8]	0.0 [0.0–0.7]	0.398
*Bifidobacteriaceae*	0.0 [0.0–0.0]	0.0 [0.0–0.3]	0.182
*Atopobium*	0.0 [0.0–0.1]	0.0 [0.0–0.0]	0.879
*Oribacterium*	0.3 [0.0–0.4]	0.0 [0.0–0.3]	0.352
*Cardiobacterium hominis*	0.0 [0.0–0.2]	0.0 [0.0–0.0]	0.447
*Bergeyella cardium*	0.2 [0.0–0.3]	0.0 [0.0–0.1]	0.193
*Catonella*	0.3 [0.0–0.4]	0.0 [0.0–0.0]	0.046
*Mogibacterium*	0.0 [0.0–0.1]	0.0 [0.0–0.0]	0.800
*Eubacterium*	0.2 [0.0–0.3]	0.0 [0.0–0.0]	0.137
*Sulfurihydrogenibium*	0.0 [0.0–1.5]	0.0 [0.0–0.0]	0.182
*Peptostreptococcus anaerobius*	0.0 [0.0–2.0]	0.0 [0.0–0.5]	0.685
*Filifactor alocis*	0.0 [0.0–0.2]	0.0 [0.0–0.0]	0.554
Other genus	1.8 [0.7–3.1]	0.6 [0.3–2.9]	0.295

**Table 6:** Cut-off values, sensitivity, specificity and area under the ROC curve for risk factors for complications after radiotherapy—RIOM.

Parameter	Cut-Off	Sensitivity	Specificity	AUC [95% CI]
*Gemella*	≥1.3%	0.375	0.906	0.764 [0.612–0.915]
*Porphyromonas*	≥6.7%	0.875	0.969	0.975 [0.928–1.000]
*Fusobacterium*	≥2.6%	0.875	0.906	0.904 [0.802–1.000]
*Catonella*	≥0.2%	0.625	0.844	0.732 [0.518–0.947]

**Table 7:** Results of univariate and multivariate logistic regression analysis for estimating the probability of complications after radiotherapy—RIOM.

Risk Factors	b	*p*	beta	*p*	OR [95% CI]
*Gemella* (%)	0.411	0.287	-	-	-
*Porphyromonas* (%)	1.089	0.013	0.860	0.013	2.97 [1.27–6.93]
*Fusobacterium* (%)	0.552	0.005	0.521	0.045	1.68 [1/08–3/61]
*Catonella* (%)	1.853	0.231	-	-	-

b—logistic correlation coefficient in univariate analysis, beta—logistic correlation coefficient in multivariate analysis, OR—odds ratio.

**Table 8:** Number (percentage) of patients in groups differing in the occurrence of RIOM and risk factors, test significance levels, and relative risks and their 95% confidence intervals.

Risk Factors	RIOM		*p*-Value	RR [95% CI]
	Yes *n* = 8	No *n* = 32		
*Gemella* ≥ 1.3%	3 (37.5%)	3 (9.4%)	0.082	3.40 [0.64–18.1]
*Porphyromonas* ≥ 6.7%	7 (87.5%)	1 (3.1%)	<0.001	28.0 [3.00–261]
*Fusobacterium* ≥ 2.6%	7 (87.5%)	3 (9.4%)	<0.001	21.0 [2.23–192]
*Catonella* ≥ 0.2%	5 (62.5%)	5 (15.6%)	0.015	5.00 [1.01–24.8]
logit model *Pr* > 0.11	7 (87.5%)	2 (6.2%)	<0.001	24.1 [2.61–223]

RR—relative risk.

## Data Availability

The raw data supporting the conclusions of this article will be made available by the authors upon request.
